# Inheritance in MGUS and MM

**DOI:** 10.18632/oncotarget.5960

**Published:** 2015-10-04

**Authors:** Michael Pfreundschuh

**Affiliations:** José-Carreras-Center for Immuno-and Gene Therapy, Dept. Internal Medicine I, Saarland University Medical School, Homburg (Saar), Germany

**Keywords:** multiple myeloma, risk factors, inheritance

Multiple myeloma (MM) is characterized by a clonal proliferation of malignant plasma cells in the bone marrow which secrete monoclonal immunoglobulins (paraproteins) into the serum. Clinical characteristics are lytic bone lesions, impairment of normal hematopoiesis and kidney function. It is accepted that all MM cases are preceded by an asymptomatic expansion of clonal plasma cells designated monoclonal gammopathy of undetermined significance (MGUS) [1]. Only a minority of individuals with MGUS evolve to symptomatic MM. The etiology of MGUS/MM is unknown and generally accepted risk factors are age > 65 years (odds ration [OR]: 12-16), male gender (OR: 1.5) and a positive family history (OR: 1.5-5.0) [2]. One hypothesis for the pathogenesis of MM is chronic antigenic stimulation; however, until recently the structures entertaining chronic antigenic stimulation remained largely unknown. To identify the antigenic targets of paraproteins (paratargs), protein macroarrays were used unmodified or after in-vitro sumoylation for screening of paraprotein-containing sera at a dilution of 1:10^7^. With unmodified macroarrays 11 autoantigens were identified as the targets of paraproteins. Of these, one was an allo-antigenic paraprotein target (sperm-specific cylicin-2 in a woman with MM), one was a heteroantigen (porcine kinesin), while the remaining nine were autoantigens [3-5]. Of the nine autoantigens all except one (where no material was available) were hyperphosphorylated in patients compared to healthy controls as the most likely reason for their autoimmunogenicity, and in all these patients the hyperphosphorylated variant was inherited as a dominant trait. While most hyperphosphorylated paratargs were found only in few families, paratarg-7 was found in 15% of European, 4.5% of Japanese, and 37(!)% of all African-American MGUS/MM patients. Due to a lower prevalence of carriers of hyperphosphorylated paratarg-7 (pP-7) in the healthy population, the OR for a healthy pP-7 carrier for MGUS/MM varies between 13.1 in the Japanese, 7.9 in the European and 4.8 in the Afro-American population. Using sumoylated macroarrays, 12% of the paraproteins from European, 11% from African-American and 5% from Japanese patients reacted specifically with sumoylated heat shock protein-90β isoform-α (HSP90-SUMO). Similar to the findings with the hyperphosphorylated paratargs, all patients with HSP90-SUMO-binding paraproteins carried HSP90-SUMO and HSP90-SUMO carrier state is inherited as an autosomal-dominant trait. HSP90-SUMO is also a strong risk factor for MGUS/MM with an OR of 14.8 in Europeans, 6.2 in Japanese and 7.4 in African-Americans [6]. With pP-7 and HSP90-SUMO taken together, roughly 30% of the European and 50% of the African-American MGUS/MM patients, respectively, carry an autosomal-dominantly inherited risk factor. Two conclusions can be drawn from these findings: 1^st^, the fact that the majority of paratargs are autoantigens with an atypical posttranslational modification as the most likely reason underlying their immunogenicity supports an important role of the modified autoantigens in the early pathogenesis of MGUS/MM by chronic autoimmunogenic stimulation; 2^nd^, assuming that many more posttranslationally modified paraprotein targets remain unidentified, we can anticipate that the majority of MGUS/MM patients is associated with an inherited risk factor.

Why was this inheritance only recently discovered and not in previous epidemiological studies? Two reasons can explain this: 1^st^, the antigenic targets of paraproteins have been discovered only recently, and 2^nd^, only a minority of carriers of posttranslationally modified autoantigens develops MGUS/MM. Thus despite the dominant inheritance of carriership of the risk factor the phenotype MGUS/MM can skip several generations and thus escape recognition in epidemiologic studies with MGUS/MM as the endpoint. That only a fraction of carriers of a modified paraprotein target develop MGUS/MM is at least in part due to the fact that at least two prerequisites must be fulfilled to develop MGUS/MM: 1^st^ carriership of the modified autoantigen, and 2^nd^, a “permissive” MHC-II haplotype, i. e. a haplotype able to present and recognize the modified autoantigenic target. This is supported by the finding that B-cells with specificity for the autoantigen need CD4^+^ T-cell help, and that only few MHC haplotypes provide such a T-cell help [7].

What are the clinical consequences of these findings? Relatives of MGUS/MM patients who are carriers of a modified autoantigen can now be easily identified by testing peripheral blood using modification-specific ELISAs. Moreover, it has recently been found that a B-cell population with specificity for the modified autoantigen can be identified which upon stimulation with the modified antigen and autologous CD4^+^ T-cells produce paraprotein-identical monoclonal antibodies (Pfreundschuh et al. submitted). This B-cell population can be targeted by toxin-conjugated paratargs and killed after internalization of the B-cell receptor / toxin-conjugate antigen complex. Thus one can now imagine to use BARs (B-cell receptor antigens for reverse targeting), either prophylactically for carriers at high risk for MGUS/MM (i. e. with a “permissive” MHC-II haplotype) or in MM patients in remission after high-dose chemotherapy and autologous stem cell transplantation to eradicate the autoantigen-specific B-cells that are the likely precursors of the malignant plasma cells and might fuel a relapse after successful induction therapy of MM.
